# Etymologia: *Toxoplasma *

**DOI:** 10.3201/eid1610.ET1610

**Published:** 2010-10

## [tok′′so-plaz′mə] 

From the Greek *tóxikon* (poisoned arrow, from *tóxon* [bow]) and *plásma* (something molded). This genus of intracellular parasitic protozoa was named for its arc-like shape. Charles Nicolle and Louis Manceaux published the definitive description of the organism in 1909 after they observed the parasites in the blood, spleen, and liver of a North African rodent, *Ctenodactylus gondii*. The species *T. gondii* was formally designated at that time.

**Source:** Ajioka JW, Morrissette NS. A century of Toxoplasma research. Int J Parasitol. 2009;39:859–60. PubMed; http://emedicine.medscape.com/article/229969-overview; Dorland’s illustrated medical dictionary, 31st edition. Philadelphia: Saunders Elsevier; 2007.

